# Innovative mobile app solution for facial nerve rehabilitation: a usability analysis

**DOI:** 10.3389/fdgth.2024.1471426

**Published:** 2024-10-21

**Authors:** Kathrin Machetanz, Mario Lins, Constantin Roder, Georgios Naros, Marcos Tatagiba, Helene Hurth

**Affiliations:** ^1^ Department of Neurosurgery and Neurotechnology, Eberhard Karls University, Tuebingen, Germany; ^2^ Institute of Networks and Security, Johannes Kepler University, Linz, Austria

**Keywords:** vestibular schwannoma, facial palsy, rehabilitation, APP, smartphone, usability

## Abstract

**Background:**

Facial palsy after vestibular schwannoma surgery is temporary in many cases but can significantly affect patients’ quality of life. Physical training—initially guided and subsequently performed by the patient—is of paramount importance for recovery of facial nerve function. The introduction of medical application software (apps) might improve therapy by maintaining motivation for daily home-based training and surveilling patients’ rehabilitation progress.

**Methods:**

We developed a mobile app, “FACEsemper”, for home-based facial nerve rehabilitation. This app guides patients through a daily training program comprising six variable exercises, each performed in three repetitions. The app allows the user to customize the exercise intensity for different facial areas and includes a reminder function for daily training. Additional features include photo documentation, a calendar function, training report generation, and the possibility of direct communication with the attending physician. The app's usability was prospectively investigated with 27 subjects, including 8 physicians, 9 patients with facial palsy and 10 healthy subjects, over a two-week period. Usability was assessed using various self-rating questionnaires (i.e., mHealth App Usability Questionnaire, MAUQ; System Usability Scale, SUS; Visual Aesthetics of Apps Inventory, VisAAI) and scores were compared across the groups.

**Results:**

The participants reported an average smartphone use of 12.19 years and completed a mean number of 290 ± 163 facial exercises during the study period. Patients used the app significantly more frequently than the other two groups (*p* = 0.017). The average total scores of the questionnaires were: MAUQ 5.67/7, SUS 89.6/100, VisAAI 5.88/7 and specific rating 6.13/7. In particular, the simplicity of use and craftsmanship of the app were rated very highly. Usability scores did not significantly differ between groups. A primary limitation identified was malfunction of the daily reminder feature in some Android versions.

**Conclusion:**

This usability study demonstrated a positive user experience and excellent usability of the FACEsemper app. However, some limitations and areas for improvement were identified. As a next step, the app should be evaluated in a large patient cohort with facial palsy to determine its potential medical benefits for facial rehabilitation compared to traditional training methods.

## Introduction

Facial palsy (FP), irrespective of its idiopathic, infectious, traumatic or iatrogenic etiology, can be an incisive life event for patients resulting in a reduced physical and mental quality of life (QoL) (1–4). Besides cause-related treatments (e.g., antiinfective therapy for bacterial or viral causes, corticosteroids), a regular training of the facial muscles is usually conducted by the patients themselves, in order to reduce the described limitations in QoL and to achieve fast facial nerve regeneration (5–7). However, missing or significantly reduced movements of the paretic side can lead to a decrease in the patient's motivation for daily, home-based training. In addition, insufficient or inadequate instructions can lead to the development of incorrect movement patterns, which might trigger an impaired healing resulting in so-called synkinesia (8).

The broad availability of smartphones and therefore also the use of medical application software (apps) enables its implementation in the field of rehabilitation and to supplement traditional treatment and training methods (9–11). Exercises can be instructed, a personalized therapy plan can be created and, most importantly training can be reminded. In addition, good accessibility of apps can help to overcome the potential shortage of physical and logopedic therapy in facial palsy caused by long distances and an inadequate number of therapists (12). Nevertheless, the training progress can be monitored by the therapists at regular intervals in addition to the use of an app.

While there are a couple of apps for grading facial palsy (13, 14), so far there are only few apps aiming to train facial function in case of a palsy, some of which offer limited options. Extensive distribution and proof of an advantage over previous therapies are completely lacking. For this reason, a specific app (FACEsemper) for training of facial palsy was developed as the fundament for this study. The aim of the present study is to evaluate the usability of the FACEsemper app before it might be used in larger patient groups. Potential malfunctions or opportunities for improvement are to be detected and the app adapted accordingly.

## Methods

### Study cohort

In total, 10 healthy subjects, 9 FP patients, and 8 physicians of the disciplines neurology, neurosurgery and plastic-reconstructive surgery gave their written informed consent and completed the study. The participating patients were recruited at the Department of Neurosurgery and Neurotechnology at the University Hospital of Tuebingen. Inclusion criterion for the patient group was the presence of a peripheral facial palsy following a tumor resection in the cerebellopontine angle (CPA). A further criterion for participation among all groups was possession of an Android smartphone, as the primary app development was performed in the Android operating system, and a transfer to the iOS operating system was planned only after usability testing and the determination of necessary changes. Exclusion criteria included treatment with Botulinum toxin in the last 6 months, facial piercings/implants and the inability to operate a smartphone independently. In addition to patients with facial palsy, healthy subjects and physicians were included in order to identify interface issues or user experience barriers that are not specific to the patient's medical condition (e.g., confusing navigation, unclear instructions) as well as to ensure that the app aligns with medical standards and therapeutic goals. For this purpose, only physicians who have regular contact with FP patients were included. The study was carried out in accordance with the recommendations of the ethics committee of the Eberhard Karls University Tuebingen (732/2021BO1) and conducted in accordance with the declaration of Helsinki.

### Software development and app functions

The FACEsemper app was designed by a software developer in close collaboration with a team of neurosurgeons from the University Hospital of Tuebingen who are familiar with postoperative facial palsies following tumor resection of the CPA. The app is built using Flutter, an open-source framework developed by Google (Google Inc., Mountain View, California, USA) (15). The programming language used within the flutter ecosystem is called Dart. One of the main advantages of using a framework like flutter is that it can be used to build multi-platform applications. That means, the majority of the codebase has to be written only once but can be deployed on different platforms like iOS or Android. However, for low-level or platform-specific functionality, native code is still required to interact with the respective interfaces. Additionally, we integrated several external libraries to provide essential functionalities such as sending notifications, playing a video, or opening a file dialog to export the training results. A full list of dependencies, including version information and license details, is available within the app.

**Figure 1:**
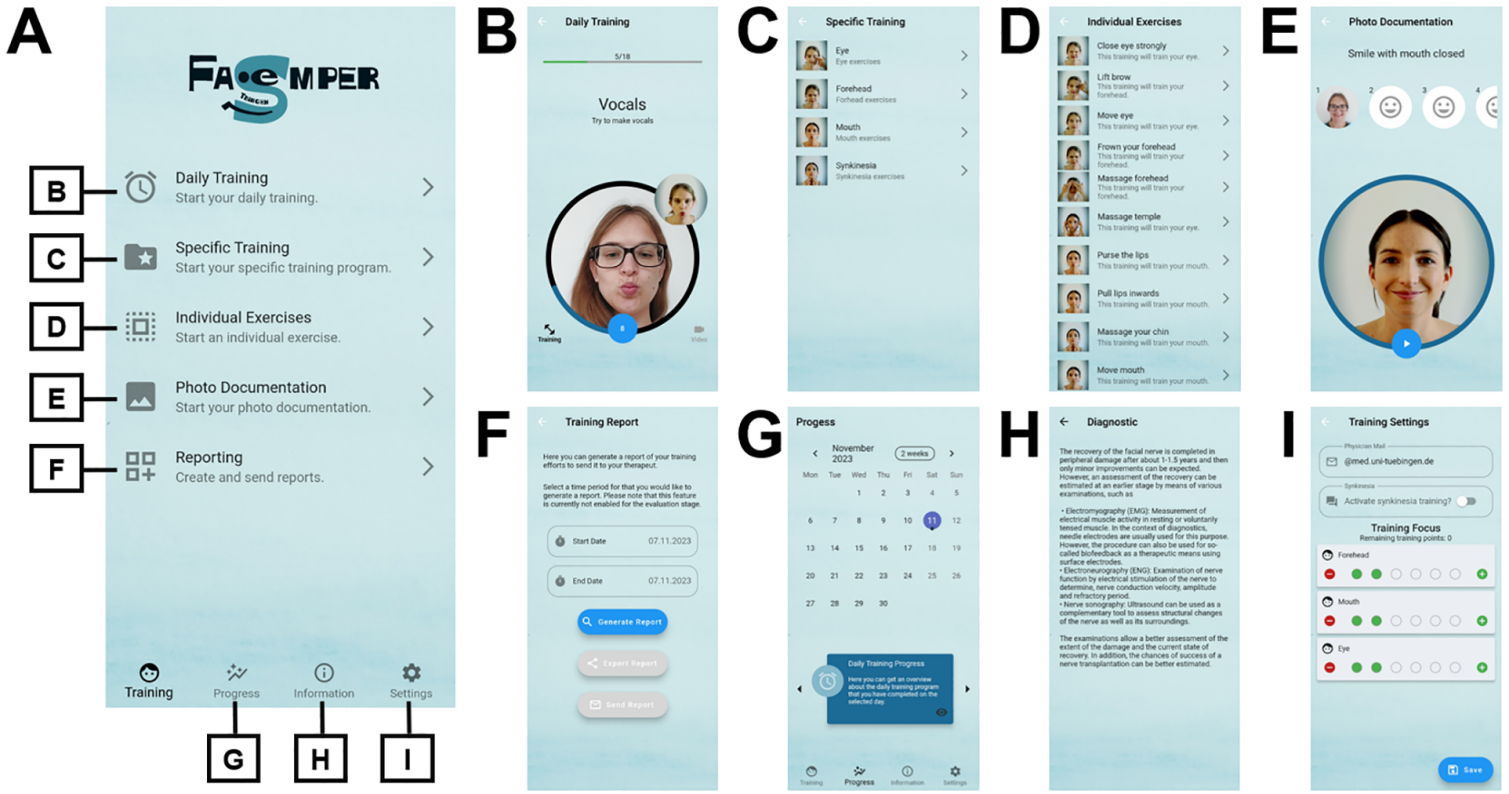
Structure of the FACEsemper app. **(A)** After initial setup, a welcome page is shown to the user. The app functionalities presented in B-I can be opened via specific buttons. **(B)** demonstrates one exercise of the daily training. In the large circle, the user's face is captured and displayed via the camera. Furthermore, video instructions for the exercise are displayed in the small circle. **(C,D)** show the specific and individual exercises. **(E)** Photo documentation is provided in a standardized form of 6 facial expressions. **(F)** Training reports can be sent for improved patient-therapist communication. **(G)** A calendrical summary showing the exercise units and photo documentation gives the user an overview of their training status. **(H)** demonstrates one of the information sides and **(I)** shows the functions of the training settings.

FACEsemper offers three training programs including daily training, programs for specific regions, and individual training sessions (Figure 1). When setting up the “*daily training*” program, the therapist and patient can determine the intensity of the training of each part of the face (i.e., forehead, eye, mouth) and whether additional synkinesia training should be performed. Based on the selected settings, various exercises for the face are randomly compiled by the app (e.g., strong eye closure, horizontal facial stretching). In the category of “*specific training*”, individual facial areas can be selected and the app creates a complete training program for the selected region. In addition, the user can choose individual exercises by using the “*individual training*” function (Figure 1).

Additional functionalities include a reminder function for the daily training, photo documentation, a calendrical overview of the training progress, the generation of a training report for a therapist and an information page where users can gain further knowledge about facial palsies approved by a medical doctor (e.g., causes or diagnostic methods). The visualization of the training progress includes both the frequency of the exercises performed and the photo documentation. Within the training report, the user can independently select which training sessions and photo documentation are sent to the therapist. Automated data transmission was not implemented to ensure patients’ privacy rights.

### Usability evaluation

In order to provide reliable evaluation, FACEsemper was tested by the participants over a period of 2 weeks. They were given the following tasks: (1) setting up the app with configuring the language and the reminder; (2) adjusting the training settings by determining the parts of the face to be trained and activating/deactivating synkinesia training; (3) performing the daily facial training for 2 weeks; (4) completing a photo documentation after each training week; (5) checking the training progress in the calendar; (6) creating and sending a training report after each training week; (7) reading the information material.

After testing and performing the aforementioned tasks, the participants completed several questionnaires to assess the usability of the FACEsemper app: mHealth App Usability Questionnaire for standalone apps (MAUQ); System Usability Scale (SUS); Visual Aesthetics of Apps Inventory (VisAAI); FACEsemper-specific evaluation questionnaire. In addition, training reports sent by the participants, were analyzed to determine how often the app was used and whether the photo documentation was completed.

The MAUQ for standalone apps is used to evaluate app functions regarding medical benefits and ease of use. It consists of a total of 18 questions, which can be assigned to the categories *ease of use* (5 items), *interface and satisfaction* (7 items) and *usefulness* (6 items). Each item can be rated from 1 (strongly disagree) to 7 (strongly agree). A mean value is calculated from all items together for the assessment (16). For this study we used the “MAUQ for Healthcare Providers” in the group of physicians and the “MAUQ for Patients” in the groups of patients and healthy subjects.

The SUS consists of 10 items and evaluates app-usability particularly in terms of ease-of-use and efficiency. The items are rated on a 5-point Likert scale from 0 (disagree) to 4 (agree). After converging the values, summing the individual items and multiplying by 2.5, the absolute SUS score can be assigned to a value of 0–100. A higher value indicates a better rating, with a value of ∼68–80 corresponding to a good rating and >80 to an excellent rating (17).

The VisAWI evaluates the aesthetics of websites with the categories *simplicity* (5 items), *diversity* (5 items), *colorfulness* (4 items) and *craftsmanship* (4 items). Each of the 18 items is rated by the user on a Likert scale from 1 (strongly disagree) to 7 (strongly agree). After re-encoding negatively polarized items, the individual scale values are added to form the scale mean of each category and then the sum is divided by the number of items in the category. The overall mean value of the VisAWI is obtained by summing all subscale values and then dividing by 4 (18). For aesthetic evaluation of the FACEsemper app we used an adjusted version of the VisAWI (VisAAI) by replacing “website” with “app” in the questionnaire.

The FACEsemper-specific questionnaire assessed the following categories of the app separately: attitudes (4 items), training (4 items for healthy subjects and patients, 5 items for physicians), calendar (3 items), photo documentation (3 items), information (2 items for healthy subjects and patients, 3 items for physicians), training report (2 items), workflow (3 items). These items could be rated by the user from 1 (strongly disagree) to 7 (strongly agree). Finally, subjects were able to give an overall assessment of the app in the categories of content, user-friendliness, aesthetics and overall impression with values from 1 (very satisfactory) to 5 (unsatisfactory) (Table 1, FACEsemper specific questionnaire *total* value) and provide open feedback.

**Table 1:** Participant characteristics.

	Overall (*n* = 27)	Physicians (*n* = 8)	Healthy subj. (*n* = 10)	Patients (*n* = 9)	
Gender					
Male	14 (51.9%)	6 (75%)	5 (50%)	3 (33%)	χ^2^ = 2.97, *P* = .28
Female	13 (48.1%)	2 (25%)	5 (50%)	6 (67%)	
Age					
18–29	7 (25.9%)	3 (37.5%)	4 (40%)	0 (0%)	χ^2^ = 13.09, ***P* = .01**
30–49	13 (48.1%)	5 (62.5%)	5 (50%)	3 (33%)	
50–65	7 (25.9%)	0 (0%)	1 (10%)	6 (67%)	
Graduation					
Mandatory school	2 (7.4%)	0 (0%)	0 (0%)	2 (22%)	χ^2^ = 9.10, ***P* = .04**
Abitur/matura	7 (25.9%)	0 (0%)	4 (40%)	3 (33%)	
University	18 (66.7%)	8 (100%)	6 (60%)	4 (44%)	
Focus of life					
City	16 (59.3%)	6 (75%)	4 (40%)	6 (67%)	χ^2^ = 3.43, *P* = .54
Suburb	3 (11.1%)	1 (12.5%)	1 (10%)	1 (11%)	
Countryside	8 (29.6%)	1 (12.5%)	5 (50%)	2 (22%)	
Employment					
Student	3 (11.1%)	0 (0%)	3 (30%)	0 (0%)	χ^2^ = 12.31, ***P* = .04**
Self-employed	3 (11.1%)	0 (0%)	2 (20%)	1 (11%)	
Employed	19 (70.4%)	8 (100%)	5 (50%)	6 (67%)	
Retired	2 (7.4%)	0 (0%)	0 (0%)	2 (22%)	
Years of smartphone use	12.19 (SD 3.09)	12.00 (SD 3.70)	11.80 (SD 2.14)	12.87 (SD 2.72)	H = 0.61, *P* = .74

Bold values mean significant results.

### Statistics

To acquire data for statistical analysis, and to create info graphics SPSS (IBM SPSS Statistics for Windows, Version 26.0. Armonk, NY: IBM Corp.) was used. An *a priori* case number calculation has been performed. To analyze nominally-scaled data we used the Chi-squared test and analyzed non-nominally distributed data employing the Kruskal-Wallis test. The data are shown as mean values (SD). *P*-values <0.05 were considered significant (confidence interval 95%). Demographic data were collected solely for descriptive statistics because of the inclusion criteria for physicians, which resulted in selection bias.

## Results

### Participant characteristics

A total of 27 subjects (i.e., 8 physicians, 9 patients with facial palsy and 10 healthy subjects) prospectively evaluated the usability of the FACEsemper app for the entire two weeks and therefore completed the study. 48.1% of participants (13/27) were female and 48.1% were between 30 and 49 years of age (13/27). Results demonstrated differences between the groups in terms of age, education level and employment status. There were no differences in age and primary residency. Moreover, participants in all groups reported using a smartphone for more than 10 years (Table 1). 40.7% of participants (11/27) have previously used a fitness app.

### Training

A total of 290 (SD 163) exercises were performed by each participant. Patients performed significantly more exercises (mean 459, SD 210) than physicians (mean 221, SD 54) and healthy subjects (mean 213, SD 40, H = 8.11, *P* = .02). During the test period, 2.2 (SD 1.8) training reports were created and 2.0 (SD 0.7) photo documentations were performed by each individual. No difference in the number of reports (H = 2.63, *P* = .27) or photo documentations (H = 1.17, *P* = .56) was found between the groups.

### Usability of the FACEsemper app

**Figure 2:**
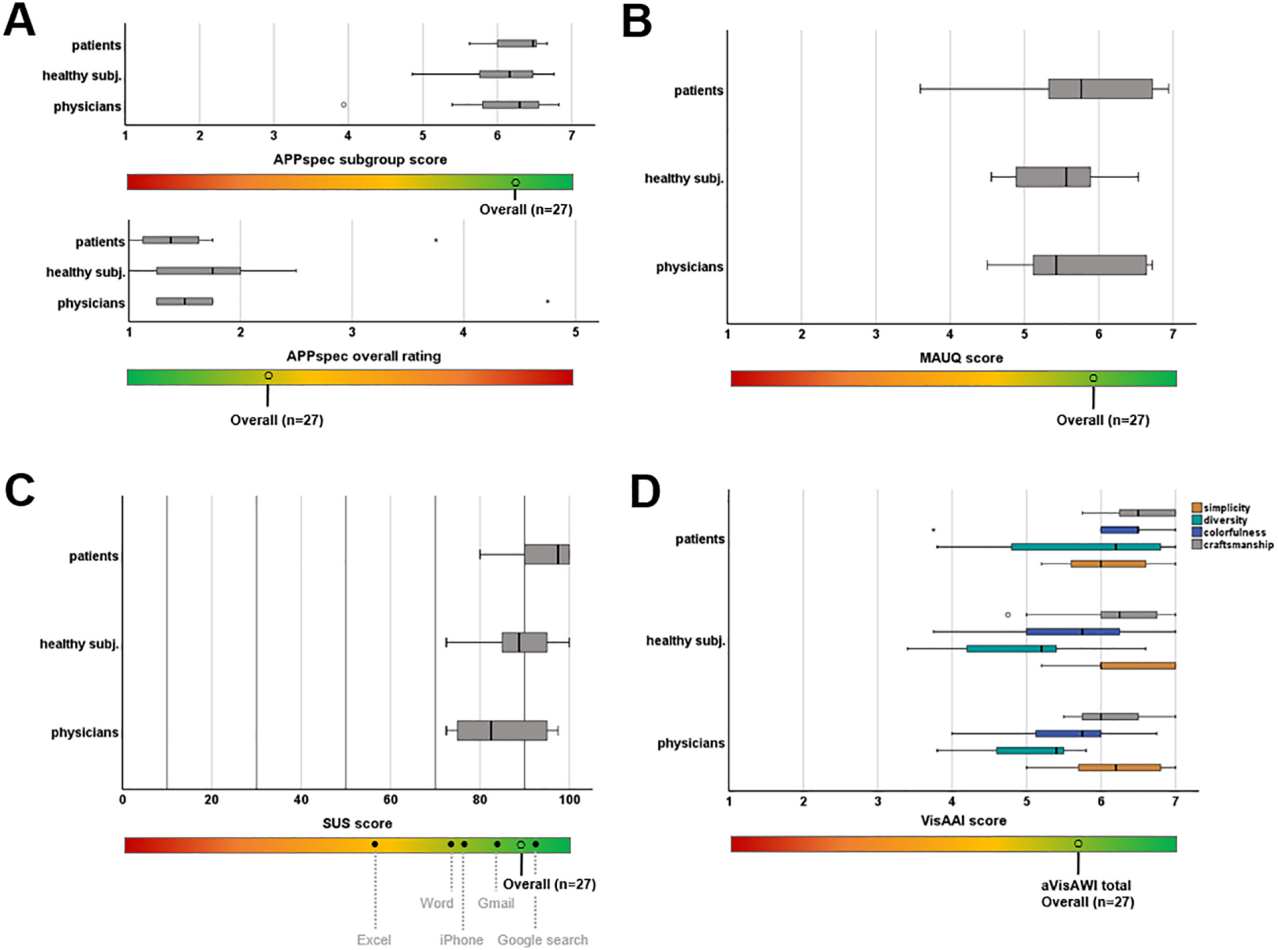
Usability evaluation by different rating scales. **(A)** FACEsemper specific questionnaire; **(B)** mHealth app Usability Questionnaire for standalone apps (MAUQ); **(C)** System Usability Scale (SUS) values are compared with SUS values of everyday products published by Kortum et al. (19); **(D)** Visual Aesthetics of Apps Inventory (VisAAI).

All participants completed the evaluation via three validated questionnaires and an app-specific questionnaire. The mean scores of the whole cohort and its subgroups are listed in Table 2. SUS scores were highest in patients and lowest in physicians. However, this difference was not statistically significant. For the VisAAI, scores between the four categories varied only slightly and showed the highest values for craftmanship (mean 6.3, SD 0.6) and lowest values for diversity (mean 5.3, SD 1.1) with no statistically significant difference between the groups of participants. Overall, the app was evaluated with good or excellent scores by all three groups (Figure 2).

**Table 2:** Usability ratings of participants.

	Overall (*n* = 27)	Physicians (*n* = 8)	Healthy subj. (*n* = 10)	Patients (*n* = 9)	
SUS (0–100)	89.6 (SD 9.3)	84.2 (SD 10.7)	88.8 (SD 8.9)	94.2 (SD 7.2)	H = 4.47, *P* = .11
VisAAI (1–7)					
Simplicity	6.2 (SD 0.7)	6.2 (SD 0.8)	6.2 (SD 0.7)	6.1 (SD 0.6)	H = 0.09, *P* = .96
Diversity	5.3 (SD 1.1)	5.0 (SD 0.8)	4.9 (SD 1.0)	5.8 (SD 1.2)	H = 2.94, *P* = .23
Colorfullness	5.8 (SD 1.0)	5.5 (SD 0.9)	5.7 (SD 1.0)	6.2 (SD 1.0)	H = 3.40, *P* = .18
Raftsmanship	6.3 (SD 0.6)	6.1 (SD 0.5)	6.2 (SD 0.8)	6.5 (SD 0.5)	H = 2.09, *P* = .35
Total	5.9 (SD 0.7)	5.7 (SD 0.5)	5.7 (SD 0.8)	6.2 (SD 0.6)	H = 2.43, *P* = .30
MAUQ (1–7)	5.7 (SD 0.9)	5.7 (SD 0.9)	5.5 (SD 0.7)	5.8 (SD 1.1)	H = 0.96, *P* = .62
FACEsemper specific questionnaire					
Subgroups (1–7)	6.1 (SD 0.7)	6.0 (SD 1.0)	6.1 (SD 0.6)	6.3 (SD 0.4)	H = 0.69, *P* = .71
Overall rating (5–1)	1.7 (SD 0.9)	1.9 (SD 1.3)	1.6 (SD 0.5)	1.6 (SD 0.9)	H = 0.85, *P* = .65

Possible scores of each questionnaire are listed within backets (worst possible–best possible score). visAAI: visual aesthetics of app inventory; MAUQ: mHealth app usability questionnaire for standalone apps; SUS: system usability scale.

### Participants’ suggestions for improvement

In total, 15/27 subjects used the opportunity to provide qualitative feedback. The analysis of the this data revealed that in particular the reminder function did not work properly. One user recommended the implementation of a reminder several times a day. Apart from a training reminder, a voluntary reminder function for creating photo documentation and the training report were suggested by several users. In addition to a visual signal, the participants also requested an acoustic signal at the end of a task so that they could receive feedback about the end of the task during exercises that involved closing their eyes. One subject asked to customize the navigation in the progress area as he did not find it intuitive. However, no specific adaptation recommendations were provided. However, the overall conclusion of the patients was positive, and it was repeatedly expressed that they would be happy to continue using the app after the end of the study. One patient wrote in this context: “By the way, I will continue to use the app. […] Overall, it helps me to practice more regularly, and the random selection of exercises is good, so there are always new stimuli”.

## Discussion

Statistical calculations suggest that 6.9 billion smartphones are in use worldwide in 2024. In Germany even 88.1% of all households are equipped with a smartphone according to the Federal Statistical Office (20). This also raises the possibility of supplementing traditional treatment methods in the medical field with the use of apps, e.g., in the rehabilitation of neurological function. Apps can provide support in different aspects: information, health habits, assessment, treatment, and specific uses (21). Previous studies in various diseases have demonstrated that the use of medical mobile applications can increase the self-efficacy of patients and at the same time, improved self-efficacy leads to an improved overall condition and physical function in patients with paresis after stroke (22–25). An improvement of rehabilitation through mobile applications is therefore also plausible for patients with FP.

In this context, the presented usability study of the FACEsemper app for training in patients with FP showed positive feedback with very good usability ratings. SUS is the most commonly used usability score to rate eHealth applications (17). Applications are considered acceptable in case of SUS scores >70 and as excellent if higher than 85 respectively (26). FACEsemper was overall rated excellent according to this scoring system. This becomes particularly obvious when comparing the SUS rating of FACEsemper in relation to everyday products such as Excel (mean SUS score 56.5, SD 18.6), Word (mean SUS score 76.2, SD 15.0) or Amazon (mean SUS score 81.8, SD 14.8) (19). Furthermore, a cut point analysis of the VisAWI showed that a score of 4.5 or higher should be aimed for new designs (27). VisAAI scores in the presented study exceeded this cut point in all subcategories and groups of participants reflecting a pleasant aesthetic design of the app. The overall rating score was 1.7, which shows a result between very satisfactory (=1) and rather satisfactory (=2).

The MAUQ questionnaire was utilized in both its patient and healthcare professional versions. The scores among patient groups and healthy individuals who completed the patient version, as well as the physicians who filled out the health professional version, were comparable. Furthermore, the physicians did not provide any suggestions for modifications or additional functions related to the app's medical content. Consequently, we conclude that these professionals, who regularly treat patients with facial palsy, offer a positive validation of the app. Moreover, the qualitative evaluation of patient feedback and the significantly more frequent use of the app by patients compared to the other two study groups suggests a genuine need for targeted features in facial rehabilitation. This higher usage could be due to patients experiencing greater benefit due to their immediate rehabilitation needs. While the study did not explicitly examine the specific motivations for higher app use by patients, it is likely that their immediate therapeutic needs led to greater engagement. Previous studies have shown in this context that there is a lack of therapeutic care for FP patients by means of specialized therapy (e.g., physiotherapy) and that 10% of patients need to travel up to more than >115 miles (=185 km) just to receive specific therapy (12, 28).

There are already other apps that have been developed for the purpose of facial training (e.g., “face2face- facial exercises”, “Face it!” and “FaceRehab”). A special feature of the face2face app is its use of an augmented reality mask during facial exercises, allowing patients to avoid constantly viewing their paretic face. This can have the advantage of maintaining motivation for practicing. On the other hand, it can also be annoying for the patient not to receive realistic feedback. The FaceRehab app, available for purchase and primarily designed for patients with Bell's palsy, offers very similar functions to the app presented (e.g., progress tracking, reminders, training plan) (29). One difference to the presented app that should be emphasized is that it contains an analysis function that determines certain viewpoints for assessing asymmetry. While this feature can provide a more objective evaluation of the rehabilitation process, it also carries the risk of patients becoming overly focused on their perceived deficits. This preoccupation can lead to demotivation, especially if they do not see immediate changes over several days or weeks. Therefore, it might be beneficial for therapists to conduct this type of analysis at designated intervals rather than having patients do it themselves. Furthermore, a) some of these apps are no longer available via the App or Play store and b) we are not aware of any studies on usability investigation or testing of an advantage compared to a therapy without the app support. Only one study by Taeger et al. (14) describes the process of developing an app, which describes similar functions/possibilities within the app, but is also not available to the public at the moment (30).

FACEsemper already includes multiple features, such as an individualized daily therapy plan, monitoring of rehabilitation progress and information about the disease (30–34). Data security and privacy aspects have also been taken into account during development, as this is an important factor in any medical care app (35, 36). Our usability evaluation demonstrated that the reminder function implemented so far is desired by patients but is still unreliable. We decided to not update the app during the study to avoid changes that could have an impact on other parts and thus potentially hinder comparison of the test results. We will resolve the issue in the next version by debugging the root cause. As recommended by the patients, we will integrate reminder functions for the photo documentation and training report as well as an acoustic signal at the end of a task so that patients could receive feedback about the end of the task. Further ideas and plans in future versions are a) to include communication with the therapist via a video consultation (in addition to the exclusive communication via the training reports), b) incorporate self-rating tools to capture the self-perceived impairment caused by facial palsy, as well as c) gamification elements (e.g., virtual coins and gadgets). Finally, the possibility of voluntary networking of FP patients will be evaluated.

### Limitations

The study's overall validity is limited by the small sample size of 27 participants, including only 9 patients with facial palsy. This may restrict the generalizability of our findings, especially as the heterogeneity of patients with facial palsy (e.g., reason and extent of facial palsy, patient's age, gender and ethnicity) was not considered in this way. A larger sample size would enhance statistical power and enable more robust analyses, which is crucial for assessing the treatment effects of the app in the future. This would help identify meaningful differences in recovery rates and improve the study's external validity. To achieve a sufficiently diverse patient population, a multi-center study involving various specialties—such as neurosurgery, neurology, ENT, and plastic surgery—would be a beneficial approach. However, previous usability studies already showed reliable results with 8–10 subjects in the assessment of the SUS questionnaire and in usability studies in general (37, 38). Furthermore, the consistent ratings in all groups indicate a usable result of the present study.

A further limitation is the fact that the data collected does not provide any information about the therapeutic benefits of the app for facial rehabilitation or the long-term usability. This is due to the test duration of two weeks, which allows evaluating the usability with regard to the described aspects such as simplicity or craftmanship as well as the basic functions of the app (e.g., photo documentation and report), but not with regard to the therapeutic effect. Future studies should extend the follow-up duration and also include a control group receiving standard physical therapy without the app to better evaluate the app's effectiveness in promoting long-term recovery and user engagement. These studies should also include objective measurement techniques to measure the facial palsy and its recovery (e.g., Sunnybrook scale or eFACE scale) (39–41).

## Conclusion

This study shows that the developed prototype of the FACEsemper app meets the requirements of a mobile application regarding usability, visual aesthetics and patient feedback was excellent. One aim of this study was to identify possible malfunctioning and detect additional necessary features which will be implemented in future FACEsemper versions. As a next step, app based facial training in addition to common therapy will be validated in a prospective randomized controlled clinical trial on patients with facial palsy of various causes. The planned follow-up study will focus on the duration until rehabilitation of the FP, the degree of rehabilitation achieved, and adherence to training over a twelve-month period. This will be compared between patients using an app-based training program combined with standard training and those using standard training alone.

## Data Availability

The datasets generated during and/or analyzed during the current study are available from the corresponding author on reasonable request.
